# Deciphering the concurrent phenomenon of childhood malnutrition by using the extended composite index of anthropometric failure (ECIAF): facts from the BESLEN project

**DOI:** 10.1017/S1368980024002520

**Published:** 2024-12-20

**Authors:** Gözde Dumlu Bilgin, İrem Kaya Cebioğlu, Hasan Kaan Kavsara, Aybüke Sarioğlu, Melis Keküllüoğlu Tan, Sema Aydin, Pınar Usta, Binnur Okan Bakir

**Affiliations:** 1 Yeditepe University, Faculty of Health Sciences, Department of Nutrition and Dietetics, İnönü Mah. Kayışdağı Cad. 34755 İstanbul, Turkey; 2 Istanbul Medipol University, Graduate School of Health Sciences, Nutrition and Dietetics, Istanbul, Türkiye

**Keywords:** Childhood malnutrition, ECIAF, Obesity, Maternal factors, Anthropometry

## Abstract

**Objective:**

To investigate the co-existence of single and multiple anthropometric failures among children using an extended composite index of anthropometric failure (ECIAF). This study aims to elucidate the complex interplay between child-specific and maternal factors, highlighting the multifaceted nature of childhood malnutrition.

**Design:**

A multicentre cross-sectional study as part of the BESLEN project

**Setting:**

Mother-Child Education Centre in the Pendik district of Istanbul, Türkiye

**Participants:**

1283 children (preschool children, *n* 822, school-aged children, *n* 462) and 1044 mothers

**Results:**

Almost 1/3 of the children included in the study had an anthropometric failure as determined by ECIAF. Weight excess was the leading cause of the total anthropometric failures, most of which were observed to be slightly higher in boys, except for stunting only and co-occurrence of stunting and underweight. Among the mother-related factors, including higher BMI and waist circumference, low maternal age at delivery, low number of children in the household and being a single parent may be considered predisposing factors to any phenomenon of childhood malnutrition. Among child-related factors, birth weight being ≥ 3500 g had a higher risk for ECIAF failure, and children aged ≥ 60 months were more likely to experience stunting and underweight, while those < 60 months had a higher prevalence of weight excess.

**Conclusions:**

The co-existence of stunting and overweight, the occurrence of weight excess in one in three stunted children and the high risk of central obesity are public health concerns. Also, ECIAF can better assess all aspects of childhood malnutrition than conventional measures.

Childhood malnutrition is a persistent global health concern, significantly impacting both individual well-being and societal development. Using anthropometric measures has long been recognised as essential for assessing nutritional status, offering a nuanced understanding of malnutrition’s diverse manifestations. Svedberg (2005) and Nandy et al. (2005) introduced the composite index of anthropometric failure (CIAF)^(1,2)^, which further expanded to address overnutrition as the extended CIAF (ECIAF) that is a notable contribution, evaluating anthropometric failures in children aged under and over 60 months^(3)^. These indices are superior to conventional measures, enabling the distinction between single and multiple anthropometric failures and guiding more targeted policy interventions^(4)^.

Undernutrition and overweight have traditionally been viewed as distinct issues with different risk factors. Poverty, food insecurity and infection were associated with undernutrition, while affluence, dietary abundance and sedentary lifestyles were linked to obesity^(5)^. However, a growing recognition emphasises that these two forms of malnutrition often coexist within communities, families and individuals, exemplified by cases of individuals experiencing both stunted growth and overweight conditions^(6)^. WHO is gradually acknowledging the significant challenge faced by low-income and middle-income countries dealing with the double burden of malnutrition^(7,8)^. This phenomenon involves the simultaneous occurrence of undernutrition, including micronutrient deficiencies, underweight, childhood stunting and wasting, alongside issues of overweight, obesity and diet-related non-communicable diseases. Current estimates indicate that globally, 2·28 billion children and adults are affected by overweight conditions, while over 150 million children experience stunted growth, underscoring the urgent need for coordinated efforts to address this dual nutritional burden^(9)^.

A growing body of evidence explores the co-existence of undernutrition and overweight in transition countries^(3,10,11)^. Findings emphasise the necessity of holistic approaches to address the double burden of malnutrition for sustainable improvements in child health parallel to Sustainable Developmental Goal 2, which aims to end all forms of malnutrition by 2030.

Shifting the focus to maternal factors, various studies have documented the influence of maternal anthropometric features on childhood malnutrition^(12,13)^. Katoch (2022) underscores the critical role of maternal nutritional status, emphasising its significant contribution to the risk of stunting and underweight in children^(14)^. This study further contributes to the existing body of evidence by reaffirming the importance of maternal anthropometrics and identifying specific parameters, such as the number of children, maternal age at delivery, marital status, and birth and feeding characteristics, as significant contributors to childhood anthropometric failures.

The objective of this study is to utilise the ECIAF to investigate the prevalence, gender-specific patterns and co-existence of single and multiple anthropometric failures among children. In addition, this study aims to explore the intricate interplay between child-specific and maternal factors, shedding light on the multifaceted nature of childhood malnutrition. The research also explores the role of child-specific factors and assesses the impact of maternal and birth-related variables, with the overarching goal of enhancing our understanding of childhood malnutrition and informing targeted public health interventions. To the best of the authors’ knowledge, this study marks the first attempt in Türkiye to use the ECIAF to determine single and multiple anthropometric failures among children under and over 60 months of age.

## Material and methods

### Participants and data collection

The BESLEN project is a multicentre cross-sectional study screening the prevalence of childhood malnutrition and examining maternal predisposing factors that may lead to malnutrition. This study aimed to reach all 1300 children and their mothers registered in the seven different Mother-Child Education Centres in the Pendik district of Istanbul, the first of the BESLEN project centres.

The data collection was carried out in two phases between October 2022 and June 2023. In the first phase, the anthropometrics of 1283 children were measured after their mothers signed the informed consent (17 children’s mothers did not volunteer to participate in any phase of the study). Subsequently, the mothers of 1044 of these children volunteered to participate in the second phase of the study in which data on the mother-related and child-related factors were collected face to face by the researchers. Apart from the overall sample, the findings were also presented by dividing the sample into two age groups: < 60 months, preschool children and ≥ 60 months school-aged children.

### Measures

The trained researchers measured children’s anthropometrics using calibrated tools in the first phase. Body weight was measured via a portable digital scale sensitive to 100 grams (g), and the standing height was measured via a portable stadiometer sensitive to 1 millimetre, with participants wearing minimum clothing. Additionally, the children’s mid-upper arm circumferences and waist circumferences (WC) were measured in centimetres (cm) with an inflexible tape, and the waist-to-height ratio (WHtR) was calculated. The prevalence of central obesity was reported in the case of children with WHtR ≥ 0·5^(15)^. The nutritional status of children was assessed using WHO criteria, and the nutritional status of preschool children was evaluated via the WHO Anthro software, while the WHO AnthroPlus software version 1.0.4 was used for school-aged children. All children were assessed and grouped according to the criteria of the CIAF and ECIAF^(3)^. Malnutrition was considered in the case of ≤ –2 sd for weight-for-height, for height-for-age and for weight-for-age. Following the WHO instructions, for school-aged children (> 60 months), the BMI-for-age was used to evaluate wasting, and malnutrition was diagnosed with a BMI-for-age ≤ –2 sd. For both age groups, the weight excess was also calculated using weight-for-height for preschoolers and BMI-for-age for school-aged children with respect to > 1 sd cut point^(3)^.

In the second phase, apart from mothers’ demographic (age, number of children, maternal age at delivery, marital, education and employment status) and anthropometric characteristics (BMI and WC), information was collected on their children’s birth (birth weight and mode of delivery) and feeding status. Only six mothers’ anthropometrics were considered invalid because they were pregnant at the data collection, so the BMI and WC measurements were presented for 1038 mothers.

Moreover, mothers completed a scale developed and validated among adults between the ages 18 and 65 years by Batmaz H. in 2018 in Türkiye named the Nutrition Knowledge Level Scale for Adults (NKLSA)^(16)^. This scale is comprised of two parts, each of which assesses a distinct aspect of adult knowledge. The first part, entitled ‘Basic Nutrition’, comprises twenty items, while the second part, ‘Food Preference’, comprises twelve items. The internal consistency of the scale is indicated by Cronbach’s *α* coefficients of 0·72 and 0·70, respectively^(16)^, which were found to be 0·78 and 0·95 in our study, respectively. Higher scores indicate higher knowledge levels.

### Statistics

The continuous variables were presented with mean and sd, and percentages were used for qualitative variables. In the first part of the study, the results were presented in two age groups: ‘< 60 months’ and ‘≥ 60 months’. The *χ* ^2^ test was used to compare the qualitative variables, and the Student *t* test or the non-parametric equivalent Mann–Whitney *U* test was used for univariate analysis of continuous outcomes. The same tests were used in the second part of the study as the results were presented in two groups regarding the children’s ECIAF status. Moreover, the OR and CI for OR were computed for failures by age groups, maternal demographics, and birth and feeding characteristics of children concerning their ECIAF status. The binary logistic regressions were performed for the significant variables to examine their effect on the likelihood of having ECIAF failure. The Hosmer–Lemeshow test and Omnibus test of model coefficients were evaluated for the goodness of fit. All statistical analyses were conducted using Statistical Package for Social Sciences (SPSS version 23, IBM) for Windows. The CI was 95 %, and findings with a *P*-value of less than 0·05 were considered statistically significant.

## Results

The findings of the study were presented and discussed in two phases. Accordingly, the first phase presented data pertaining to children, and the second phase included data pertaining to the mothers.

### Findings of the first phase

The mean age of the 1283 children (preschool children, *n* 822, school-aged children, *n* 462) was 57·9 (sd 6·5) months, of which 48·2 % were girls and 51·8 % were boys. Considering the anthropometrics, the mean WC was 54·4 (sd 5·1) cm, and the mean mid-upper arm circumference was 17·5 (sd 1·9) cm among all children, and the results of these two measurements were quite similar between the two age groups. However, the WHtR was slightly but significantly higher among preschool children (*P* < 0·001) (Table 1).

**Table 1.** Demographic and anthropometric characteristics of children (Numbers and percentages; mean values and standard deviations)

		Overall (*n* 1283)			< 60 months (*n* 822, 64·1 %)			≥ 60 months (*n* 461, 35·9 %)			
		*n*		%	*n*		%	*n*		%	
Sex	Boys	664		51·8	414		50·4	250		54·2	
	Girls	619		48·2	408		49·6	211		45·8	
		Mean	sd	Min.–Max.	Mean	sd	Min.–Max.	Mean	sd	Min.–Max.	
Age (months)		57·9	6·5	41–84	53·9	3·5	41–59	65·1	4·2	60–84	
Weight (kg)		19·2	3·5	10·8–39·2	18·7	3·2	10·8–39·2	20·2	3·7	13·1–37·2	
Height (cm)		109·3	5·7	89–128	107·5	5·2	89·0–126·0	112·3	5·5	95·0–128·0	
MUAC (cm)		17·5	1·9	11–27	17·5	1·9	11–27	17·6	2·03	12–24	
WC (cm)		54·4	5·1	40–104	54·2	5·0	40–104	54·8	5·2	43·0–85·5	
											*P* ^a^
WHtR		0·5	0·04	0·4–1·03	0·50	0·04	0·4–1·03	0·49	0·04	0·4–0·8	< 0·001**

Min, minimum; Max. maximum; MUAC, mid-upper arm circumference; WC, waist circumference; WHtR, waist-to-height ratio.

^a^Independent sample *t* test.

**P < 0·01.

**Fig. 1:**
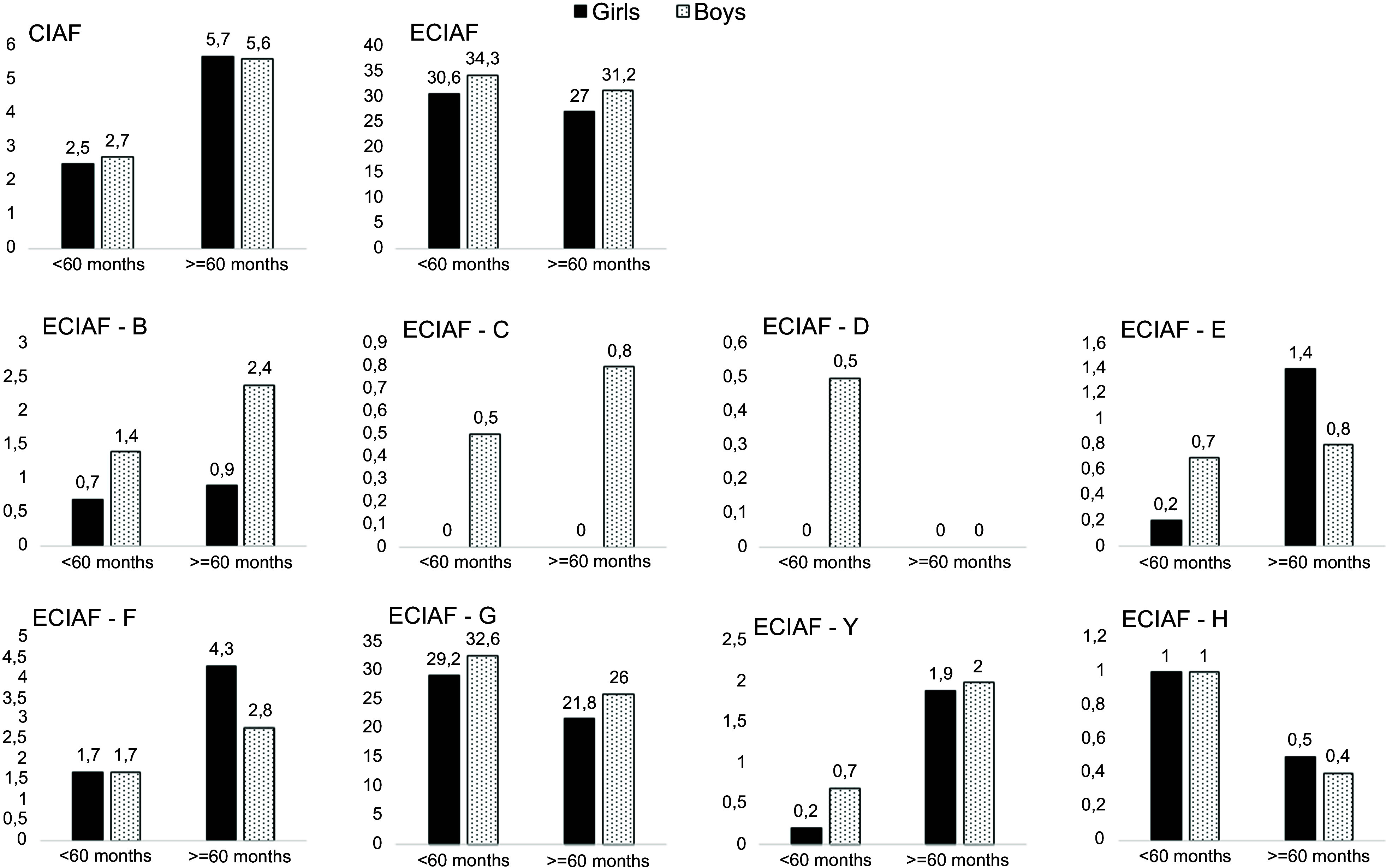
CIAF and ECIAF failure for age groups by sex. CIAF, composite index of anthropometric failure; ECIAF, extended composite index of anthropometric failure; CIAF – A, no failure for CIAF; ECIAF – A, B, wasting only; C, wasting and underweight; D, stunting, wasting and underweight; E, stunting and underweight; F, stunting only; Y, underweight only; G, weight excess (overweight or obese) only; H, stunting and weight excess.

As seen in Table 2, the anthropometric failure prevalence determined by CIAF among overall children was 3·7 %, while it was 31·3 % by ECIAF. The prevalence of CIAF among preschool children was 2·6 %, even significantly higher among school-aged children, with a prevalence of 5·6 % (*P* < 0·01). However, the failure prevalence was remarkably higher for both age groups considered by ECIAF: 32·5 % for preschoolers and 29·3 % for school-aged children. When the prevalence distribution was rearranged regarding sex, it was observed that boys had a higher prevalence of anthropometric failure by ECIAF in preschoolers and school-aged children (34·3 % and 31·2 %, respectively) (Fig. 1).

**Table 2.** Prevalence of CIAF and ECIAF with different forms of anthropometric failures (Numbers and percentages; odds ratios and 95 % confidence intervals)

	Overall (*n* 1283)		< 60 months		≥ 60 months				
	%	*n*	%	*n*	%	*n*	*P*	OR	95 % CI
CIAF (B + C + D + E + F + Y)	3·7	47	2·6	21	5·6	26	0·005^a^^**^	2·28	1·27, 4·10
ECIAF (B + C + D + E + F + Y + G + H)	31·3	402	32·5	267	29·3	135	0·236^a^	1·16	0·91, 1·51
(A) No failure for CIAF	96·3		97·4		94·4		–	–	
(A) No failure for ECIAF	68·7		67·5		70·7		–	–	
(B) Wasting only	1·3	17	1·1	9	1·7	8	0·336^a^	1·6	0·6, 4·2
(C) Wasting and underweight	0·3	4	0·2	2	0·4	2	0·622^b^	1·8	0·25, 12·7
(D) Stunting, wasting and underweight	0·2	2	0·2	2	0		NA	NA	
(E) Stunting and underweight	0·7	9	0·5	4	1·1	5	0·296^b^	2·24	0·60, 8·4
(F) Stunting only	2·3	30	1·7	14	3·5	16	0·044^a^^*^	2·10	1·0, 4·3
(Y) Underweight only	1·0	13	0·5	4	2·0	9	0·018^b^^*^	4·10	1·25, 13·3
(G)^c^ Weight excess (overweight or obese) only	28·4	365	30·9	254	24·1	111	0·009^a^^*^	1·41	1·1, 1·8
(H)^c^ Stunting and weight excess	0·8	10	1·0	8	0·4	2	0·510^b^	2·3	0·5, 10·7

CIAF, composite index of anthropometric failure; ECIAF, extended composite index of anthropometric failure; NA, not applicable.

^a^Pearson’s *χ*^2^ test.

^b^Fisher’s exact test.

^c^Parameters only included for ECIAF.

*P < 0·05.

**P < 0·01.

All categories for single failures were observed to be more prevalent among school-aged children than preschoolers, except category G (weight excess). Wasting (category B) was determined with a 1·3 % prevalence among all children, while it was 1·1 % for preschoolers. Although not statistically significant, it was found that the odds of being wasted were 1·6 times higher among school-aged children than among preschoolers (CI 0·6, 4·2), with a prevalence of 1·7 %. Regarding sex distribution, it was observed that the prevalence of wasting was twice as high among preschool boys, while it was even higher among school-aged boys than among girls (Fig. 1). The prevalence of stunting (category F) was 2·3 % for all children, 1·7 % for preschoolers and significantly higher at 3·5 % for school-aged children (*P* < 0·05), with odds of 2·10 (CI 1·0, 4·3). Contrary to other categories, stunting was more prevalent among school-aged girls than boys, with a prevalence of 4·3 % and 2·8 %, respectively, but equal among preschoolers (Fig. 1). Underweight (category Y) prevalence was also 1 % among all children, which was doubled for school-aged children (2·0 %, *P* < 0·05). In addition, school-aged children were 4·1 times more likely to be underweight than preschool children (CI 1·25, 13·3). The failure Y was more prevalent among boys than girls in both age groups (Fig. 1). According to the data, 28·4 % of all children were diagnosed with category G (weight excess only). Contrary to the prevalence of other single failures, preschool children had a significantly higher prevalence of being overweight or obese than school-aged children at 30·9 %. They are 1·41 times more likely to be overweight or obese (CI 0·52, 1·17). In addition, in both age groups, boys were observed to have a higher prevalence of weight excess failure than girls (Fig. 1).

Considering the simultaneous dual failures, the prevalence was higher among school-aged children for categories C (wasting + underweight) and E (stunting + underweight) than preschoolers (for category C, 0·4 % and 0·2 % and for category E, 1·1 % and 0·5 %, respectively). On the contrary, 1·0 % of preschoolers had a simultaneous dual failure of category H (stunting + weight excess), while it was 0·4 % of school-aged children. Additionally, no school-aged children were determined to be at risk for triple failure (category D), but this category has a 0·2 % prevalence among preschoolers. All children identified in categories C and D consisted of boys (Fig. 1). The dual failure of stunting + underweight was more prevalent among school-aged girls than boys, but this prevalence was more common among preschool boys (Fig. 1).

The anthropometrics of the children were also considered regarding their ECIAF status. As shown in Table 3, children who have ECIAF failure have significantly higher mean WC (57·8 cm, sd 6·2), higher mean WHtR (0·53, sd 0·05) and higher mid-upper arm circumference (18·8 cm, sd 2·1) (*P* < 0·001 for all).

**Table 3.** Anthropometric measurements regarding the ECIAF status of the children (Mean values and standard deviations)

	ECIAF						
	Failure (*n* 402)			No failure (*n* 881)			
	Mean	sd	Min.–Max.	Mean	sd	Min.–Max.	*P*
WC (cm)	57·8	6·2	43–104	52·9	3·4	40·0–85·5	< 0·001^a^^**^
WHtR	0·53	0·05	0·38–1·03	0·49	0·03	0·4–0·8	< 0·001^a^^**^
MUAC (cm)	18·8	2·1	13–26	16·9	1·5	11–24	< 0·001^b^^**^

ECIAF, extended composite index of anthropometric failure; Min, minimum; Max, maximum; WC, waist circumference; WHtR, waist-to-height ratio; MUAC, mid-upper arm circumference.

^a^Independent sample *t* test.

^b^Mann–Whitney *U* test.

^c^Parameters only included for ECIAF.

**P < 0·01.

### Findings of the second phase

The mean age of 1044 mothers was 34 (sd 5·6) years, ranging from 21 to 56. In terms of anthropometric measures, the mean BMI was 28·0 (sd 5·6) kg/m^2^, indicating overweight, and the range was quite wide, from 15·9 to 60·2 kg/m^2^. On the other hand, the WC falls within the desired range with a mean of 87·7 (sd 12·9) cm. Moreover, there was statistical significance in the ECIAF status of their children; BMI and WC were significantly higher in mothers of children with at least one failure (*P* < 0·001 for both) (Table 4).

**Table 4.** Demographic, anthropometric and scores of nutrition knowledge level of mothers (Mean values and standard deviations; numbers and percentages; odds ratios and 95 % confidence intervals)

	ECIAF									
	Overall (*n* 1044)			Failure (*n* 306)			No failure (*n* 738)			
	Mean	sd	Min.-Max.	Mean	sd	Min.-Max.	Mean	sd	Min.-Max.	*P* ^a^
Age (years)	34	5·6	21–56	33·5	5·5	21–47	34·2	5·6	21–56	0·052
BMI (kg/m^2^) (*n* 1038)	28·0	5·6	15·9–60·2	29·2	5·7	17·2–51·8	27·5	5·5	15·9–60·2	< 0·001**
WC (cm) (*n* 1038)	87·7	12·9	53–136	90·1	13·0	57–127	86·7	12·7	53–136	< 0·001**
Number of children	2·3	0·9	1–7	2·2	0·9	1–6	2·3	1·0	1–7	0·021*
Maternal age at delivery (years)	28·4	5·7	16–52	27·8	5·6	17–41	28·7	5·8	16–52	0·040*
Basic nutrition score	44·6	10·9	17–71	43·9	11·2	20–71	44·8	10·9	17–70	0·235
Food preference score	30·3	12·9	0–48	29·4	13·0	0–48	31·0	12·8	0–48	0·147

		%		*n*	%		*n*	%		*n*	*P* ^*b*^	OR	95 % CI
Marital status	Currently married	98·3		1026	28·8		295	71·2		731	0·003**	3·89	1·5, 10·1
	Single	1·7		18	61·1		11	38·9		7			
Education level	Illiterate	1·3		14	28·6		4	71·4		10	0·982	NA	
	Literate	1·6		17	23·5		4	76·5		13			
	Primary school	22·7		237	30		71	70		166			
	Middle school	20·7		216	29·6		64	70·4		152			
	High school	35·2		368	28·3		104	71·7		264			
	University/College	18·4		192	30·7		59	69·3		133			
Employment status	Unemployed/Housewife	95·6		998	29		289	71		709	0·244	1·44	0·8, 2·7
	Employed	4·4		46	37		17	63		29			

ECIAF, extended composite index of anthropometric failure; Min, minimum; Max, maximum; WC, waist circumference.

^a^Independent sample *t* test.

^b^Pearson’s *χ*^2^ test.

*P < 0·05.

**P < 0·01.

As presented in Table 4, the mean number of children of the mothers was 2·3 (sd 0·94), ranging from 1 to 7. This mean was the same for the ECIAF no failure group, however slightly but significantly lower for the ECIAF failure group (*P* < 0·05). A similar pattern was observed for the maternal age at delivery with a mean of 28·4 (sd 5·7) years, almost the same as in the ECIAF no failure group, but mothers of children in the ECIAF failure group had a significantly lower maternal age at delivery (*P* < 0·05).

The vast majority of the women (98·3 %) were married at the time of data collection, and 95·6 % of them were unemployed/housewives, although more than half of them reported having a high school or university degree (35·2 % and 18·4 %, respectively). Regarding the ECIAF status of their children, no statistically significant risk was observed for their employment or educational status (*P* > 0·05). However, it was observed that the odds of having ECIAF failure in children were significantly increased by 3·89 (CI 0·5, 10·1) for single mothers (*P* < 0·01) (Table 4).

In the second part of the study, the mothers’ nutrition knowledge level was also examined and presented in Table 4 in two parts. Accordingly, the mean total basic nutrition score of mothers was 44·6 (sd 10·9), with a range from 17 to 71, and the mean total food preference score was 30·3 (sd 12·9), ranging from 0 to 48. These scores did not differ significantly between ECIAF groups (*P* > 0·05 for both).

In Table 5, the birth and feeding characteristics of children were presented. Of the 1044 children, 1015 (97·2 %) were breastfed, and the mean duration of breast-feeding was 19·3 (sd 9·5) months, ranging from 0·5 to 60 months. However, breastfed children were slightly but insignificantly more likely to have ECIAF failure (29·4 %, *P* > 0·05). Moreover, although a slightly longer duration of breast-feeding was reported in children without ECIAF failure, this difference was not significantly different (*P* > 0·05). The proportion of formula users among children was 43·2 %, and 28·8 % of formula users had ECIAF failure compared with 29·7 % of non-users. However, this difference was insignificant (*P* > 0·05).

**Table 5.** Birth and feeding characteristics of children (Numbers and percentages; mean values and standard deviations; odds ratios and 95 % confidence intervals)

		ECIAF											
		Overall (*n* 1044)			Failure			No failure					
		%		*n*	%		*n*	%		*n*	*P* ^a^	OR	95 % CI
Breastfed status	Breastfed	97·2		1015	29·4		298	70·6		717	0·836	1·1	0·5, 2·5
	Not Brestfed	2·8		29	27·6		8	72·4		21			
Formula use	Formula used	43·2		451	28·8		130	71·2		321	0·764	0·96	0·7, 1·3
	No Formula	56·8		593	29·7		176	70·3		417			
Mode of delivery	Vaginal	44·9		469	28·6		134	71·4		335	0·636	1·1	0·8, 1·4
	C-section	55·1		575	29·9		172	70·1		403			
Birth weight categories	< 3500 g	62·5		653	26·3		172	73·7		481	0·006	1·5	1·1, 1·9
	≥ 3500 g	37·5		391	34·3		134	65·7		257			

	Mean	sd	Min.–Max.	Mean	sd	Min.–Max.	Mean	sd	Min.–Max.	*P* ^b^
Duration of breastmilk (months) (*n* 1015)	19·3	9·5	0·5–60	18·9	9·7	0·5–60	19·5	9·4	1–60	0·386
Birth weight (g)	3229·4	624·5	630–5200	3288·4	629·2	630–4900	3204·9	621·4	700–5200	0·049*

ECIAF, extended composite index of anthropometric failure; Min, minimum; Max, maximum.

^a^Pearson’s *χ*^2^ test.

^b^Independent sample *t* test.

*P < 0·05.

The proportion of children born by cesarean section was 55·1 %, of whom 29·9 % had ECIAF failure, compared with a slightly but insignificantly lower rate of 28·6 % in children born vaginally (*P* > 0·05). When the birth weight of the children was analysed, the mean of overall 1044 children was 3229·4 (sd 624·5) kg which were quite similar in groups according to the ECIAF failure status of the children. However, although the mean birth weights of children with and without ECIAF failure were so close and fell within the clinically normal birth weight range, this small difference was statistically significant, indicating that those with ECIAF had a higher birth weight with a mean of 3288·4 (sd 629·2) kg (*P* < 0·05). Due to the similar means between the two groups of ECIAF, this significant finding was investigated further. Accordingly, the cut-off value was determined as 3500 g^(17,18)^ and accordingly, it was determined that children with a birth weight above this value had a 1·5-fold higher risk of ECIAF (Table 5).

Binary logistic regression was conducted for the variables significantly related to children’s ECIAF status. There was one categorical explanatory variable, marital status, and two continuous explanatory variables, maternal BMI and WC, for the binary logistic regression model to explain the factors contributing to children’s ECIAF status. Accordingly, the binary logistic regression results showed that maternal BMI, number of children and marital status of the mother significantly affected the likelihood of having ECIAF failure (Table 6).

**Table 6.** Contributions of some maternal factors to the ECIAF status of the children

	B	se	Wald	df	*P*	OR	95 % CI
Maternal BMI	−0·47	0·021	4·825	1	0·028*	0·955	0·916, 0·995
Maternal WC	−0·008	0·009	0·737	1	0·391	0·992	0·974, 1·010
Number of children	0·170	0·083	4·181	1	0·041*	1·186	1·007, 1·396
Maternal age at delivery	0·023	0·013	2·940	1	0·086	1·023	0·997, 1·051
Marital status							
Married	–	–	–	–	–	–	–
Single	−1·239	0·513	5·841	1	0·016*	0·290	0·106, 0·791
Test	χ2	df	*P*				
Hosmer and Lemeshow	11·098	8	0·196				
Omnibus likelihood ratio	38·355	5	0·000				

ECIAF, extended composite index of anthropometric failure; B, coefficient for the constant; df, degree of freedom; WC, waist circumference.

*P < 0·05.

## Discussion

Determining the prevalence of malnutrition in communities is important to increase targeted interventions to reduce malnutrition globally, in line with the 2030 Sustainable Development Goals^(19)^. As is common in transition countries, Türkiye has undergone structural changes over decades, and obesity, along with malnutrition, has become a public health concern. Several studies also emphasised obesity as an alarming risk, with an 11·6-fold increase in prevalence over the last 20 years in our country^(10,20)^. Moreover, according to a recent report by WHO (2022), Türkiye has been listed as the tenth in obesity and overweight prevalence among WHO European region countries, with a total prevalence of nearly 50 %^(21)^. This study is noteworthy because it is the first to use the ECIAF index to assess both overnutrition and undernutrition in children under and over 60 months of age.

Almost one in three children included in the study had an anthropometric failure determined by ECIAF. This finding falls within the range identified among Ghanaian children by Kuwornu et al. (2022)^(4)^, although another study data of 10 879 children from six different regions of Argentina indicated a lower prevalence (15·1 %)^(3)^ and also a higher prevalence (37·5 %) was reported from El Salvador conducted in 334 school children^(22)^. When looking at the sex and age groups, this prevalence shows a similar distribution, although it is slightly higher in boys, in line with the literature^(3,22)^, and also in preschoolers. Contrarly, Bejarano et al. (2019) also indicated a lower prevalence among boys with ages lower than 60 months^(3)^. When all ECIAF categories were evaluated, weight excess categories (overweight and obesity) were found to be the main contributor to total anthropometric failure, similar to other studies^(3,22)^. In addition, more than half of the children, which is higher than the literature^(22)^, had an elevated risk of central obesity on the basis of their WHtR, and this risk was 6·11 times higher in children with ECIAF failure, in support of the findings from ECIAF. A recent review of the 25-year trend in overweight and obesity in Türkiye reported a doubling of the prevalence in preschool children, from 6·6 % to 14·4 %^(11)^. The current study reported an even higher prevalence (28·4 %) in both age groups, which once again underlines the increasing trend in the prevalence of obesity. Supportively, studies reported high levels of obesity in children from households with a lower socio-economic level in high-income countries and children living in urban areas in low- and middle-income countries^(11)^. It should be highlighted that this study was conducted in a low socio-economic area of the country’s largest metropolitan city, home to diverse demographic groups.

Stunting, wasting and underweight failures were the most common single failures after the category of weight excess among overall children, indicating the risk of chronic and acute malnutrition as well as obesity in the population. The co-existence of overnutrition and undernutrition phenomenon can be explained by the double burden of malnutrition, which is common in transition countries^(3,10,11)^; although Türkiye is not listed among them, this phenomenon persists as a public health concern in our country.

Among the single failures following category G (weight excess), undernutrition (wasting, stunting and underweight) was more prevalent in school-aged children than in preschoolers, parallel to the literature^(23)^. Moreover, the odds of being stunted were 2·10 times, and being underweight 4·1 was significantly higher among school-aged children. This trend could be elucidated by an increase in energy consumption due to being physically more active and the persistence of poor nutrition practices, which are an extension of related maternal and childcare factors. In terms of sex distribution, the only single failure is stunting, which is higher among girls, particularly those of school age, contrary to the literature^(24)^. However, wasting and underweight were higher in boys than in girls, in keeping with the various studies indicating that malnutrition/retarded growth (stunting + underweight) was more prevalent in boys^(22–25)^. This observation has been attributed to the female eco-stability hypothesis, which states that girls’ smaller body size and naturally greater adiposity confer an advantage in being more resistant to external environmental factors that modulate ontogenetic development^(25,26)^. The multiple anthropometric failure prevalence of overall children was not at a remarkable level in our study. However, the prevalence of simultaneous existence of stunting and weight excess was at a comparable level to Pedrero et al. findings (2022), indicating 0·9 % ECIAF-H failure^(22)^. Moreover, based on the findings that ten out of thirty stunted children were overweight/obese in this study, we should emphasise that one in every three stunted children has a condition related to weight excess. This observation is supported by the literature, as short stature is considered a notable factor in the obesity epidemic among children and adults^(3)^. Furthermore, children with multiple failures are more prone to have ill health and an increased mortality risk^(4)^.

Besides the environmental and genetic factors that contribute to childhood malnutrition, the impact of parental influence should not be overlooked. In many societies, mothers are generally the primary caregivers of children and make an important contribution to children’s healthy eating patterns. Thus, understanding the role of maternal factors is of great importance in controlling childhood malnutrition.

Current anthropometric characteristics of mothers have been identified as one of the strongest predictors of malnutrition levels in children, especially excess weight^(27)^. Our study findings also confirmed that BMI and WC are significantly higher in mothers of children with ECIAF failure. Parallel to the present findings, a study by Vehapoğlu et al. revealed that mothers who were overweight were significantly more likely to have obese children compared with normal-weight mothers^(28)^. Another study has reported that the BMI of the mother can account for 25–40 % of the BMI of the children^(29)^. This effect may be attributed to heavier mothers’ lower levels of physical activity, their preference for high-fat foods and their less healthy food choices^(30)^.

The mother’s education level and employment status may be counted as potential contributors to childhood obesity; this study could not display any statistical significance regarding the anthropometric failures of children. Interestingly, although single mother population was quite low in this study, a remarkable finding has demonstrated that the risk of ECIAF failure in children of single mothers was significantly increased by 3·89 times. Consistently, several researchers have pointed out that children of single parents are more likely to be obese^(31,32)^. Single-parent households’ lack of resources, preference for foods high in saturated fat and sugar, low levels of physical activity, lack of time to prepare healthy meals, increased exposure to television and uncertain housing conditions can be cited as potential arguments for this association^(11,33)^. Moreover, we observed a significantly lower maternal age at delivery among the ECIAF group, similar to a study among the Chinese population indicating maternal age under 28 years as a remarkable factor in predicting childhood obesity^(34)^.

Maternal feeding practices have been described as an important variable contributing to childhood obesity, and approaches may differ between primiparous and multiparous mothers^(35)^. The present study revealed that mothers’ mean number of children was significantly lower in the ECIAF group. Consistent with this outcome, several researchers have documented higher obesity prevalence in children born to primiparous mothers compared with children born to multiparous women^(36,37)^. The concept of parity and childhood obesity can be mediated by sibling interactions, higher physical activity opportunities and the tendency for household income to be shared equally among all children, which reduces access to large amounts of food and/or increases the need for restrictions in large families^(38)^.

Considering the processes that begin with the birth of the child, it is reported that infant feeding practices, as well as mode of delivery and birth weight, may have an impact on childhood obesity. Several studies reported the protective effect of prolonged breast-feeding practices on children’s risk of obesity^(27,39)^. However, in addition to the limitations of the study designs, environmental, genetic, cultural and maternal and infant variables have all contributed to the inconsistent findings in the literature regarding the effect of breast-feeding on obesity^(40,41)^. Thus, this study failed to observe any statistical significance in relation to the ECIAF status of the children and their breast-feeding status, duration and formula use, supporting the literature. Regarding the mode of delivery, WHO recommends no more than 10–15 per cent of the population to have C-sections; over the past few years, there has been a dramatic increase in global C-section rates, including Türkiye, which is among the top five countries with the highest rate (50·8 %) in the world^(42,43)^. Although this study did not determine any statistical difference regarding ECIAF failure of the children, a mounting body of literature supports a relationship between C-sections and an increased risk of overweight and obesity in offspring^(34,44,45)^. Birth weight predominantly reflects intra-uterine developmental conditions, particularly maternal–fetal nutrition, and recently, the ‘fetal origins hypothesis’ has emerged as one of the most potent theoretical frameworks in medicine regarding the early causes of later disease^(46)^. Supportingly, a substantial body of research is evidence that high birth weight is a risk factor for childhood obesity^(47,48)^. Consistent with this observation, the current study has demonstrated that children with ECIAF had significantly higher birth weights. One of the possible explanations might be related to increased levels of prenatal growth hormones, including insulin and insulin-like growth factors I and II, which can increase the risk of obesity later in life^(48)^.

A recent study has demonstrated that low levels of maternal nutritional knowledge are associated with an elevated risk of childhood obesity^(49)^. Although we hypothesised that children of mothers with high nutritional knowledge would have a lower prevalence of ECIAF, the study indicates no significant association between obesity and mothers’ knowledge of basic nutrition and their food preferences, despite their awareness of the relationship between diet and health, in line with Forh et al. (2022) study^(50)^. This circumstance justifies the fact that knowledge does not always translate into practice and that a number of potential confounding variables, including availability, accessibility, utilisation and stability of food, may influence the situation.

To highlight the strengths of this study, a high number of children were reached, as well as the simultaneous collection of data from the mothers of these children enabled the findings to be obtained through realistic observations of measures. Moreover, we had the chance to obtain anthropometric data objectively via calibrated devices by trained researchers. Despite these strengths, the cross-sectional design of the study limits the determination of the causal relationship between variables. Also, another handicap is that data on birth-related factors of children are collected based on the mother’s memory. Moreover, although ECIAF evaluates all aspects of the child’s anthropometry, insufficient reflection of micronutrient deficiencies precludes the evaluation of the triple burden of malnutrition.

## Conclusion

This is the first study in Türkiye that used the ECIAF to determine the concurrent phenomenon of malnutrition, including single and multiple anthropometric failures among children under and over 60 months of age, and investigated the possible contributors associated with child-specific and maternal factors.

According to the findings of this study, we can conclude that weight excess was the main contributor to total anthropometric failures, most of which were observed to be slightly higher in boys except stunting only and co-occurrence of stunting and underweight. Moreover, the simultaneous existence of stunting and overweight, the observation that one in every three stunted children has a condition related to weight excess, and the elevated central obesity risk can be counted as concerning public health issues. This study also contributes an important aspect by investigating the role of maternal and birth-related factors in controlling childhood malnutrition. Accordingly, among the mother-related factors, including higher BMI and WC, low maternal age at delivery, low number of children in the household and being a single parent may be considered predisposing factors to any phenomenon of childhood malnutrition. Among child-related factors, birth weight being 3500 g and over, had a higher risk for ECIAF failure, and children aged 60 months or older were more likely to experience stunting and underweight, while those under 60 months had a higher prevalence of weight excess.

In conclusion, it is possible to assess all aspects of childhood malnutrition better using ECIAF rather than conventional measures, making it a promising tool for both future research and clinical practice.

## References

[1] Svedberg P (2000) Poverty and Undernutrition: Theory, Measurement, and Policy. (Oxford, 2000; online edn, Oxford Academic). Oxford: Oxford University Press.

[2] Nandy S , Irving M , Gordon D et al. (2005) Poverty, child undernutrition and morbidity: new evidence from India. Bull World Health Organ 83, 210–216. 15798845 PMC2624218

[3] Bejarano IF , Oyhenart EE , Torres MF et al. (2019) Extended composite index of anthropometric failure in Argentinean preschool and school children. Public Health Nutr 22, 3327–3335. 31640824 10.1017/S1368980019002027 PMC10260443

[4] Kuwornu JP , Amoyaw J , Manyanga T et al. (2022) Measuring the overall burden of early childhood malnutrition in Ghana: a comparison of estimates from multiple data sources. Int J Heal Policy Manag 11, 1035–1046. 10.34172/ijhpm.2020.253 PMC9808187 33589568

[5] Doak CM , Adair LS , Bentley M et al. (2005) The dual burden household and the nutrition transition paradox. Int J Obes 29, 129–136. 10.1038/sj.ijo.0802824 15505634

[6] Wells JC , Sawaya AL , Wibaek R et al. (2020) The double burden of malnutrition: aetiological pathways and consequences for health. Lancet 395, 75–88. 31852605 10.1016/S0140-6736(19)32472-9 PMC7613491

[7] World Health Organisation (2017) The Double Burden of Malnutrition. https://iris.who.int/bitstream/handle/10665/255413/WHO-NMH-NHD-17.3-eng.pdf?ua=1 (accessed 15 December 2023).

[8] Alem AZ , Yeshaw Y , Liyew AM et al. (2023) Double burden of malnutrition and its associated factors among women in low and middle income countries: findings from 52 nationally representative data. BMC Public Health 23, 1–16. 37537530 10.1186/s12889-023-16045-4 PMC10398981

[9] Popkin BM , Corvalan C & Grummer-Strawn LM (2020) Dynamics of the double burden of malnutrition and the changing nutrition reality. Lancet 395, 65–74. 31852602 10.1016/S0140-6736(19)32497-3 PMC7179702

[10] Comba A , Demir E & Barış Eren N (2019) Nutritional status and related factors of schoolchildren in Çorum, Turkey. Public Health Nutr 22, 122–131. 30406743 10.1017/S1368980018002938 PMC10260469

[11] Görçin Karaketir Ş , Lüleci NE , Eryurt MA et al. (2023) Overweight and obesity in preschool children in Turkey: a multilevel analysis. J Biosoc Sci 55, 344–366. 35086578 10.1017/S0021932022000025

[12] Amaha ND & Woldeamanuel BT (2021) Maternal factors associated with moderate and severe stunting in Ethiopian children: analysis of some environmental factors based on 2016 demographic health survey. Nutr J 20, 1–9. 33639943 10.1186/s12937-021-00677-6 PMC7916293

[13] Mamidi RS , Banjara SK , Manchala S et al. (2022) Maternal nutrition, body composition and gestational weight gain on low birth weight and small for gestational age—a cohort study in an Indian Urban Slum. Children 9, 1460. 36291396 10.3390/children9101460 PMC9600910

[14] Katoch OR (2022) Determinants of malnutrition among children: a systematic review. Nutrition 96, 111565. 35066367 10.1016/j.nut.2021.111565

[15] Hsieh SD , Yoshinaga H & Muto T (2003) Waist-to-height ratio, a simple and practical index for assessing central fat distribution and metabolic risk in Japanese men and women. Int J Obes 27, 610–616. 10.1038/sj.ijo.0802259 12704405

[16] Batmaz H (2018) Development of A Nutrition Knowledge Level Scale For Adults and Validation Reliability Study. Graduate Thesis, Marmara University, İstanbul. https://toad.halileksi.net/wp-content/uploads/2022/07/yetiskinler-icin-beslenme-bilgi-duzeyi-olcegi-gelistirilmesi-ve-gecerlik-guvenirlik-calismasi-toad.pdf (accessed 15 December 2023).

[17] Laopaiboon M , Lumbiganon P , Rattanakanokchai S et al. (2019) An outcome-based definition of low birthweight for births in low- and middle-income countries: a secondary analysis of the WHO global survey on maternal and perinatal health. BMC Pediatr 19, 1–9. 31132994 10.1186/s12887-019-1546-z PMC6535858

[18] Shi J , Guo Q , Fang H et al. (2024) The relationship between birth weight and the risk of overweight and obesity among Chinese children and adolescents aged 7–17 years. Nutrients 16, 715. 38474841 10.3390/nu16050715 PMC10935436

[19] United Nations (2014) Report of the Open Working Group of the General Assembly on Sustainable Development Goals. http://www.un.org/ga/search/view_doc.asp?symbol=A/68/970&Lang=E (accessed 15 December 2023).

[20] Alper Z , Ercan İ & Uncu Y (2018) A meta-analysis and an evaluation of trends in obesity prevalence among children and adolescents in Turkey: 1990 through 2015. JCRPE J Clin Res Pediatr Endocrinol 10, 59–67. 28901943 10.4274/jcrpe.5043 PMC5838374

[21] World Health Organisation (2022) European Regional Obesity Report 2022. Copenhagen: World Health Organisation.

[22] Pedrero-Tomé R , López-Ejeda N , Sánchez Alvarez M et al. (2022) Household food insecurity and nutritional status of schoolchildren in rural regions of Bajo Lampa, El Salvador (2018–2019). Ecol Food Nutr 61, 128–143. 34428106 10.1080/03670244.2021.1968851

[23] Berhanu A , Garoma S , Arero G et al. (2022) Stunting and associated factors among school-age children (5–14 years) in Mulo district, Oromia region, Ethiopia. SAGE Open Med 10, 1–10. 10.1177/20503121221127880 PMC9536101 36212231

[24] Thurstans S , Opondo C , Seal A et al. (2020) Boys are more likely to be undernourished than girls: a systematic review and meta-analysis of sex differences in undernutrition. BMJ Glob Heal 5, e004030. 10.1136/bmjgh-2020-004030 PMC7745319 33328202

[25] Díez Navarro A , Marrodán Serrano MD , Gómez De Arriba A et al. (2017) Female eco-stability and severe malnutrition in children: Evidence from humanitarian aid interventions of Action Against Hunger in African, Asian and Latin American countries. Nutr Clin Diet Hosp 37, 127–134.

[26] Stini WA (1975) Adaptive strategies of human populations under nutritional stress. In Biosocial Interrelations in Population Adaptation, pp. 19–41 [ ES Watts , FE Johnston and GW Lasker , editors]. The Hague: Mouton.

[27] Ma J , Qiao Y , Zhao P et al. (2020) Breastfeeding and childhood obesity: a 12-country study. Matern Child Nutr 16, 1–9. 10.1111/mcn.12984 PMC7296809 32141229

[28] Vehapoglu A , Goknar N , Turel O et al. (2017) Risk factors for childhood obesity: do the birth weight, type of delivery, and mother’s overweight have an implication on current weight status? World J Pediatr 13, 457–464 28434072 10.1007/s12519-017-0030-9

[29] Anderson PM & Butcher KF (2006) Childhood obesity: trends and potential causes. Future Child 16, 19–45. 10.1353/foc.2006.0001 16532657

[30] Fuemmeler BF , Lovelady CA & Zucker NL (2013) Parental obesity moderates the relationship between childhood appetitive traits and weight. Obesity (Silver Spring) 21, 815–823. 23712985 10.1002/oby.20144 PMC3671382

[31] Duriancik DM & Goff CR (2019) Children of single-parent households are at a higher risk of obesity: a systematic review. J Child Heal Care 23, 358–369. 10.1177/1367493519852463 31129999

[32] Huffman FG , Kanikireddy S & Patel M (2010) Parenthood-a contributing factor to childhood obesity. Int J Environ Res Public Health 7, 2800–2810. 20717539 10.3390/ijerph7072800 PMC2922726

[33] Hsu PC , Hwang FM , Chien MI et al. (2022) The impact of maternal influences on childhood obesity. Sci Rep 12, 1–6. 35428792 10.1038/s41598-022-10216-w PMC9012806

[34] Liu S , Lei J , Ma J et al. (2020) Interaction between delivery mode and maternal age in predicting overweight and obesity in 1123 Chinese preschool children. Ann Transl Med 8, 474. 32395518 10.21037/atm.2020.03.128 PMC7210148

[35] Hu S , Wacharasin C & Sangin S (2023) Factors associated with feeding behaviors among mothers of obese infants: a cross-sectional study. Transl Pediatr 12, 1004–1016. 37305717 10.21037/tp-23-185 PMC10248945

[36] Woronko C , Merry L , Uckun S et al. (2023) Prevalence and determinants of overweight and obesity among preschool-aged children from migrant and socioeconomically disadvantaged contexts in Montreal, Canada. Prev Med Rep 36, 102397. 37732020 10.1016/j.pmedr.2023.102397 PMC10507148

[37] Gaillard R , Rurangirwa AA , Williams MA et al. (2014) Maternal parity, fetal and childhood growth, and cardiometabolic risk factors. Hypertension 64, 266–274. 24866145 10.1161/HYPERTENSIONAHA.114.03492

[38] Park SH & Cormier E (2018) Influence of siblings on child health behaviors and obesity: a systematic review. J Child Fam Stud (Internet) 27, 2069–2081.

[39] Qiao J , Dai LJ , Zhang Q et al. (2020) A meta-analysis of the association between breastfeeding and early childhood obesity. J Pediatr Nurs 53, 57–66. 32464422 10.1016/j.pedn.2020.04.024

[40] Grube MM , Von Der Lippe E , Schlaud M et al. (2015) Does breastfeeding help to reduce the risk of childhood overweight and obesity? A propensity score analysis of data from the KiGGS study. PLoS One 10, e0122534. 25811831 10.1371/journal.pone.0122534 PMC4374721

[41] Lefebvre CM & John RM (2014) The effect of breastfeeding on childhood overweight and obesity: a systematic review of the literature. J Am Assoc Nurse Pract 26, 386–401. 24170411 10.1002/2327-6924.12036

[42] World Health Organisation (2015) *WHO Statement on Caesarean Section Rates*. Geneva: World Health Organisation. https://iris.who.int/bitstream/handle/10665/161442/WHO_RHR_15.02_eng.pdf?sequence=1 (accessed 15 December 2023).

[43] Betran AP , Ye J , Moller AB et al. (2021) Trends and projections of caesarean section rates: global and regional estimates. BMJ Glob Heal 6, e005671. 10.1136/bmjgh-2021-005671 PMC8208001 34130991

[44] Li HT , Zhou YB & Liu JM (2013) The impact of cesarean section on offspring overweight and obesity: a systematic review and meta-analysis. Int J Obes 37, 893–899. 10.1038/ijo.2012.195 23207407

[45] Zhang S , Qin X , Li P et al. (2022) Effect of elective cesarean section on children’s obesity from birth to adolescence: a systematic review and meta-analysis. Front Pediatr 9, 793400. 35155315 10.3389/fped.2021.793400 PMC8829565

[46] Schellong K , Schulz S , Harder T et al. (2012) Birth weight and long-term overweight risk: systematic review and a meta-analysis including 643 902 persons from 66 studies and 26 countries globally. PLoS One 7, e47776. 23082214 10.1371/journal.pone.0047776 PMC3474767

[47] Qiao Y , Ma J , Wang Y et al. (2015) Birth weight and childhood obesity: a 12-country study. Int J Obes 5, S74–S79. 10.1038/ijosup.2015.23 PMC4850624 27152189

[48] Yu ZB , Han SP , Zhu GZ et al. (2011) Birth weight and subsequent risk of obesity: a systematic review and meta-analysis. Obes Rev 12, 525–542. 21438992 10.1111/j.1467-789X.2011.00867.x

[49] Xu Z , Zhao Y , Sun J et al. (2022) Association between dietary knowledge and overweight and obesity in Chinese children and adolescents: evidence from the China Health and Nutrition Survey in 2004–2015. PLoS One 17, 1–14. 10.1371/journal.pone.0278945 PMC9733866 36490274

[50] Forh G , Apprey C & Frimpomaa Agyapong NA (2022) Nutritional knowledge and practices of mothers/caregivers and its impact on the nutritional status of children 6–59 months in Sefwi Wiawso Municipality, Western-North Region, Ghana. Heliyon 8, e12330. 36590498 10.1016/j.heliyon.2022.e12330 PMC9798164

